# Treatment of nonmetastatic castration-resistant prostate cancer: focus on second-generation androgen receptor inhibitors

**DOI:** 10.1038/s41391-020-00310-3

**Published:** 2021-02-08

**Authors:** Fred Saad, Martin Bögemann, Kazuhiro Suzuki, Neal Shore

**Affiliations:** 1 Department of Urology, Centre Hospitalier de l’Université de Montreal (CHUM), Montreal Cancer Institute/CRCHUM, Montreal, QC Canada; 2 Department of Urology, Münster University Medical Center, Münster, Germany; 3 Department of Urology, Gunma University Graduate School of Medicine, Gunma, Japan; 4 Carolina Urologic Research Center, Atlantic Urology Clinics, Myrtle Beach, SC USA

**Keywords:** Urological cancer, Cancer

## Abstract

**Background:**

Nonmetastatic castration-resistant prostate cancer (nmCRPC) is defined as a rising prostate-specific antigen concentration, despite castrate levels of testosterone with ongoing androgen-deprivation therapy or orchiectomy, and no detectable metastases by conventional imaging. Patients with nmCRPC progress to metastatic disease and are at risk of developing cancer-related symptoms and morbidity, eventually dying of their disease. While patients with nmCRPC are generally asymptomatic from their disease, they are often older and have chronic comorbidities that require long-term concomitant medication. Therefore, careful consideration of the benefit–risk profile of potential treatments is required.

**Methods:**

In this review, we will discuss the rationale for early treatment of patients with nmCRPC to delay metastatic progression and prolong survival, as well as the factors influencing this treatment decision. We will focus on oral pharmacotherapy with the second-generation androgen receptor inhibitors, apalutamide, enzalutamide, and darolutamide, and the importance of balancing the clinical benefit they offer with potential adverse events and the consequential impact on quality of life, physical capacity, and cognitive function.

**Results and conclusions:**

While the definition of nmCRPC is well established, the advent of next-generation imaging techniques capable of detecting hitherto undetectable oligometastatic disease in patients with nmCRPC has fostered debate on the criteria that inform the management of these patients. However, despite these developments, published consensus statements have maintained that the absence of metastases on conventional imaging suffices to guide such therapeutic decisions. In addition, the prolonged metastasis-free survival and recently reported positive overall survival outcomes of the three second-generation androgen receptor inhibitors have provided further evidence for the early use of these agents in patients with nmCRPC in order to delay metastases and prolong survival. Here, we discuss the benefit–risk profiles of apalutamide, enzalutamide, and darolutamide based on the data available from their pivotal clinical trials in patients with nmCRPC.

## Introduction

**Fig. 1:**
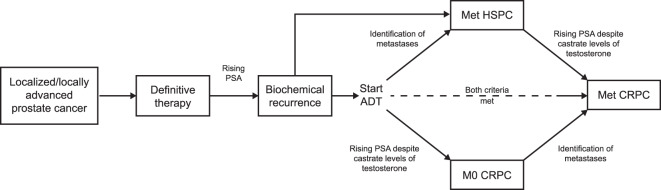
The course of prostate cancer. Reproduced with permission from Anantharaman A, Small EJ. Tackling nonmetastatic castration-resistant prostate cancer: special considerations in treatment. *Expert Rev Anticancer Ther*. 2017. ADT androgen-deprivation therapy, CRPC castration-resistant prostate cancer, HSPC hormone-sensitive prostate cancer, M0 nonmetastatic, Met metastatic, PSA prostate-specific antigen.

Since the establishment of prostate-specific antigen (PSA) screening in the 1980s, prostate cancer incidence rates have risen: ~1.3 million men are diagnosed each year and up to 20% of men present with radiographic evidence of metastases at diagnosis [1, 2]. However, after primary treatment, localized or locally advanced disease may progress to biochemical recurrence, sometimes termed “biochemical failure,” usually within 5 years [3]. If salvage surgery or radiotherapy is not an option or is ineffective, androgen-deprivation therapy (ADT), via chemical or surgical castration with gonadotropin-releasing hormone agonists or antagonists, or orchiectomy, represents the standard of care, but most patients eventually fail to maintain suppression of PSA levels [1, 4]. The mechanisms for this have been ascribed to dysregulated androgen signaling through gain-of-function mutations, splice variants, aberrant post-receptor regulation, and intratumoral androgen synthesis [5]. Such abnormal signaling contributes to neoplastic cellular proliferation and tumor progression to the more lethal forms of the disease [6]. Castration-resistant prostate cancer (CRPC) is defined by a castrate serum testosterone level <50 ng/dL with either biochemical or radiological progression [1, 7]. In patients with CRPC, metastases detected by conventional imaging with computerized tomography (CT) or technetium-99m scintigraphy are defined as metastatic castration-resistant prostate cancer (mCRPC), whereas CRPC without radiographic evidence of metastases is categorized as nonmetastatic CRPC (nmCRPC) [1, 8]. The course of prostate cancer is summarized in Fig. 1.

In 2011, Kirby et al. published a systematic review of 12 epidemiological studies on a total of 71,179 patients observed for up to 12 years [9]. Despite their inherent heterogeneity, four of five prevalence studies suggested that 10–20% of patients with prostate cancer develop CRPC during approximately 5 years of follow‐up [9]. In the United States (US), the annual incidence of nmCRPC has been estimated at approximately 60,000 men in 2020, with an annual progression rate to metastatic disease of 34% and an annual overall mortality of 16% [10]. A quantitative assessment model of US populations most at risk for disease progression found that while mCRPC may arise from either metastatic hormone-sensitive prostate cancer or nmCRPC, a significant proportion of patients progress from the nmCRPC state [10].

This review discusses the rationale for early treatment of patients with nmCRPC to delay metastatic progression and ultimately prolong survival. We will focus on treatment of nmCRPC with second-generation androgen receptor inhibitors (ARIs), the importance of balancing the clinical benefit of these treatments with potential adverse events (AEs), and the consequential impact on health-related quality of life (HRQoL), physical capacity, and cognitive function.

## Diagnosing nmCRPC in clinical practice

The most widely adopted definition of nmCRPC, derived from the recommendations of the Prostate Cancer Working Group 3, is a 25% increase from the PSA nadir (considering an initial value of ≥1 ng/mL) in men with castrate levels of serum testosterone, with a minimum rise of 2 ng/mL, which must be confirmed with a second value obtained 1–3 weeks later without evidence of metastases [11].

Various imaging techniques are routinely utilized to stage prostate cancer and to detect metastases and tumor recurrence. The National Comprehensive Cancer Network (NCCN) Clinical Practice Guidelines for Prostate Cancer (v1.2020) recommend CT or magnetic resonance imaging (MRI) for staging with the addition of conventional bone scans for the detection of metastases [4]. Next-generation techniques, such as positron emission tomography (PET)/CT or PET/MRI, are only recommended to address equivocal findings. The guidelines note that conventional bone scans are rarely positive for asymptomatic men with PSA < 10 ng/mL. However, since the risk of metastases or death increases as PSA doubling time (PSADT) shortens, skeletal scintigraphy should be performed more frequently when PSADT is ≤8 months [4]. The European Association of Urology (EAU) guidelines on prostate cancer (2020) recommend that a conventional bone scan and CT scan should be performed when PSA is 2 ng/mL in asymptomatic patients [7]. If the scans are negative, they should be repeated when PSA is 5 ng/mL, and again after every doubling of the PSA. Symptomatic patients should undergo relevant investigation, regardless of PSA level [8].

While the Surveillance, Epidemiology, and End Results database validation of the NCCN recommendations confirmed the reliability and utility of conventional bone scans, which would have missed only 0.14% of patients with bone metastases, the utility of next-generation imaging techniques in nmCRPC is under investigation [12]. Fendler et al. retrospectively screened 8825 patient records to determine detection rates by prostate-specific membrane antigen-PET (PSMA-PET) for pelvic disease and distant metastases (M1) in high-risk cases of nmCRPC on prior conventional MRI/CT [13]. Two-hundred patients (2.27%) satisfied the definition of high risk with PSADT ≤ 10 months on continuous ADT and/or a Gleason score ≥8. Patients underwent either PSMA-PET or PET/MRI. Overall, PSMA-PET was positive in 98% of the 200 study patients, of whom 44% had disease confined to the pelvis and 55% had M1 disease [13]. These findings may suggest that in the clinical studies of patients with nmCRPC and PSADT ≤ 10 months, specifically the PROSPER, SPARTAN, and ARAMIS studies (discussed below), a subgroup of patients may have developed micrometastases that were not detected at baseline with conventional imaging. However, it is important to note that patients in these studies still experienced clinical benefit in terms of delaying metastases and prolonging survival, independently of the potential presence of microscopic tumor foci [14–16].

Although PET/CT imaging with ^68^Ga (gallium)- or ^18^F (fluoride)-PSMA has been adopted in parts of Europe and Australia, current guidelines do not endorse the use of these techniques beyond diagnosis or biochemical relapse. In addition, in many cases, the cost of PSMA-based PET imaging is not reimbursed [17].

In terms of clinical opinion, both the 2019 Advanced Prostate Cancer Consensus Conference (APCCC) and a Canadian-based consensus forum found that the vast majority of the panel would treat a patient who had a PSADT < 10 months and no metastases by conventional imaging with an agent approved for nmCRPC, regardless of whether PET-based imaging was positive for metastases [18, 19]. Furthermore, at the 2019 APCCC, consensus on the use of PET-based imaging to guide treatment decisions in patients with nmCRPC was not achieved [19]. Until future clinical trials can demonstrate an advantage of staging with such techniques (in terms of clinical outcomes such as metastasis-free survival [MFS] and overall survival [OS] in patients with nmCRPC), CT, MRI, and/or technetium-99m scintigraphy for the evaluation of distant, soft tissue, or skeletal metastases in patients with nmCRPC and rising PSA levels remain the standard of care and are readily accessible. Indeed, the three second-generation ARIs approved for the treatment of nmCRPC that we discuss in this review were assessed in pivotal clinical trials that adopted conventional imaging to confirm the absence of metastases in patients with PSADT ≤ 10 months [14–16]. Thus, the current utility of next-generation imaging techniques to guide treatment decisions for patients with nmCRPC in clinical practice remains limited, and patients with rising PSA levels and no metastases on conventional imaging will continue to be classified as “nonmetastatic.”

## Rationale for treating nmCRPC

Progression of nmCRPC to metastatic disease is likely to involve lymph nodes and/or bone; approximately one-third of patients will develop osseous metastasis within 2 years, most commonly involving sites in the pelvis, spine, or ribs [20, 21]. Among patients with nmCRPC, a higher PSA concentration at diagnosis, shorter PSADT, higher Gleason score, a history of primary intervention, and a shorter interval from ADT initiation to the diagnosis of CRPC, have been associated with shorter time to metastasis [22]. It is generally accepted that poor prognosis of patients with nmCRPC is most closely suggested by shorter PSADT [23]. Following the development of metastases, the prognosis for OS diminishes [24].

Patients with bone metastases are at significant risk of developing symptomatic skeletal events (SSEs); history of an SSE is considered a negative predictor of survival [25]. The association of SSEs with mortality might be explained by the presence of advanced disease, declining performance status, loss of independence, and pathological fractures [25]. While SSEs cause pain and adversely impact quality of life, their management is associated with significant healthcare resource utilization, which has been found to be consistent in global, multinational studies [26, 27].

Effective management of nmCRPC should aim to delay metastatic progression and subsequent initiation of antineoplastic therapy, while also attempting to prolong survival and maintain quality of life [8, 28]. Ideally, treatment should be initiated as early as possible after development of nmCRPC in patients at the greatest risk of progression, i.e., those with a PSA level >2 ng/mL and a PSADT of ≤10 months [10].

While delaying the onset of metastases in patients with nmCRPC is an important treatment goal, there are several factors that require careful consideration when initiating pharmacotherapy, specifically age, underlying comorbidities, associated concomitant medications, and symptoms induced by chronic ADT. Patients with nmCRPC are described as generally asymptomatic from their disease, and while this is true from a tumor burden perspective, it does not reflect the entire clinical composite [29, 30]. The risk of developing prostate cancer increases with age and the majority of patients diagnosed with prostate cancer are older [31, 32]. Thus, the probability of developing prostate cancer is 4.7% in men aged 60–69 years, and 8.2% in those aged ≥70 years, but just 1.8% in men aged 50–59 years [31]. Older patients may experience physical impediments often ascribed to sarcopenia (i.e., loss of skeletal muscle mass and function) and fatigue related to comorbidities, including depression, anemia, hypotension, hypothyroidism, and vitamin B_12_ deficiency [33].

In addition, age-related comorbidities that require long-term, concomitant medication, are common in patients with prostate cancer, particularly cardiovascular conditions, such as hyperlipidemia, hypertension, arrhythmias, and congestive heart failure [34, 35]. As a result, the most frequently used medications among patients with prostate cancer are statins, antiplatelet agents, angiotensin-converting enzyme inhibitors, β-blockers, and calcium channel blockers. Nonsteroidal anti-inflammatory drugs and nonopioid analgesics are also frequently taken by these patients [34, 35]. Further, as patients with nmCRPC are likely to receive continuous ADT, the associated hormonal depletion may induce or exacerbate adverse effects on cognitive function, cardiovascular health, sexual health, insulin sensitivity, and bone health (including falls and fractures) [36].

Given the multifactorial aspects of the aging process that intersect in this disease state, it is essential that clinicians balance the therapeutic benefits of ARIs against the risk of treatment-emergent AEs and potential drug–drug interactions [37], thereby ensuring that quality of life and the ability to perform independent activities of daily living are preserved among patients with nmCRPC.

## Second-generation ARIs for nmCRPC: benefit–risk considerations

Despite biochemical recurrence in patients treated with definitive first-line ADT, the androgen receptor (AR) remains an important driver of prostate cancer and, thus, the primary target of pharmacologic intervention. So-called “first-generation” ARIs, such as bicalutamide, were the first steroidal analogs to demonstrate successful inhibition of the AR [38]. However, acquired resistance to these agents commonly occurred due to AR amplification, point mutations, expression of ligand-independent AR splice variants, intratumoral androgen production, or downstream signaling mechanisms [38]. Prior to the availability of second-generation ARIs, addition of these first-generation ARIs to ADT was common, despite the lack of significant efficacy, and they continue to be prescribed in clinical practice, possibly due to drug cost, access, or familiarity with these agents. In recent years, second-generation ARIs have been developed that inhibit AR function not only by blocking the ligand-binding domain, as with ADT, but also by preventing nuclear translocation and interaction of the dimerized AR with DNA [14, 15, 39].

Apalutamide, a second-generation oral ARI, was approved by the FDA in 2018 for the treatment of nmCRPC [40]. Apalutamide directly interacts with the ligand-binding domain of the AR, inhibiting translocation of the activated receptor complex to the nucleus, and subsequent binding to DNA [41]. Drug approval was based on efficacy demonstrated in the primary analysis of the phase 3 SPARTAN trial of apalutamide versus placebo (both with ADT) in patients with nmCRPC and a PSADT ≤ 10 months [15]. At the time of the primary analysis (median follow-up: 20.3 months), apalutamide was associated with a significant increase in median MFS compared with placebo (40.5 vs. 16.2 months, *P* < 0.001), and reduced the risk of distant metastasis or death by 72% versus placebo (hazard ratio [HR] 0.28; 95% CI 0.23–0.35, *P* < 0.001) [15]. In the final analysis of the SPARTAN trial (median follow-up: 52.0 months), apalutamide was associated with significantly prolonged median OS compared with placebo (73.9 vs 59.9 months; HR 0.78; 95% CI 0.64–0.96, *P* = 0.016), and improvements in times to initiation of cytotoxic chemotherapy, symptomatic progression, PSA progression, and second disease progression [42]. The SPARTAN trial and its treatment outcomes are summarized in Table 1.

**Table 1:** Summary of pivotal trials for second-generation ARIs in nmCRPC.

	SPARTAN (NCT01946204) [15, 42, 61]	PROSPER (NCT02003924) [14, 45, 62]	ARAMIS (NCT02200614) [16, 48, 63]
Study description	Randomized phase 3 study to evaluate the safety and efficacy of apalutamide vs. placebo in patients with nmCRPC (*N* = 1207)	Randomized phase 3 study to evaluate the safety and efficacy of enzalutamide vs placebo in patients with nmCRPC (*N* = 1401)	Randomized phase 3 study to evaluate the safety and efficacy of darolutamide vs placebo in patients with nmCRPC (*N* = 1509)
Patient population	nmCRPC with PSADT ≤ 10 months	nmCRPC with BL PSA ≥ 2 ng/mL and PSADT ≤ 10 months	nmCRPC with PSADT ≤ 10 months
	No lesions detectable by CT/MRI or bone scan	No lesions detectable by CT/MRI and bone scan and no history of seizure	No lesions detectable by CT/MRI or bone scan
**Primary analysis**			
Median follow-up	20.3 months	Enzalutamide: 18.5 months Placebo: 15.1 months	17.9 months
Primary endpoint (drug vs. placebo)	Median MFS, assessed from randomization until radiographic progression by blinded independent central review or death	Median MFS, assessed from randomization until radiographic progression by blinded independent central review or death	Median MFS, assessed from randomization until radiographic progression by blinded independent central review or death
	40.5 vs. 16.2 months; HR 0.28; 95% CI 0.23–0.35; *P* < 0.001	36.6 vs. 14.7 months; HR 0.29; 95% CI 0.24–0.35; *P* < 0.001	40.4 vs. 18.4 months; HR 0.41; 95% CI 0.34–0.50; *P* < 0.001
Secondary endpoints evaluated in a hierarchical order (drug vs. placebo)	Median PFS: 40.5 vs. 14.7 months; HR 0.29; 95% CI: 0.24–0.36; *P* < 0.001	Median time to PSA progression: 37.2 vs. 3.9 months; HR 0.07; 95% CI 0.05–0.08; *P* < 0.001	Median OS: NR vs. NR; HR 0.71; 95% CI 0.50–0.99; *P* = 0.045
	Median time to symptomatic progression: NR vs. NR; HR 0.45; 95% CI 0.32–0.63; *P* < 0.001	Median time to first use of new antineoplastic therapy: 39.6 vs. 17.7 months; HR 0.21; 95% CI 0.17–0.26; *P* < 0.001	Median time to pain progression: 40.3 vs. 25.4 months; HR 0.65; 95% CI 0.53–0.79
	Median OS: NR vs. 39.0 months; HR 0.70; 95% CI 0.47–1.04; *P* = 0.07	Median OS: NR vs. NR; HR 0.80; 95% CI 0.58–1.09; *P* = 0.15	Median time to first use of cytotoxic chemotherapy: NR vs. 38.2 months; HR 0.43; 95% CI 0.31–0.60
	Median time to first cytotoxic chemotherapy: NR vs. NR; HR 0.44; 95% CI 0.29–0.66		Median time to first SSE: NR vs. NR; HR 0.43; 95% CI 0.22–0.84
			Median PFS: 36.8 vs. 14.8 months; HR 0.38; 95% CI 0.32–0.45
			Median time to PSA progression: 33.2 vs. 7.3 months; HR 0.13; 95% CI 0.11–0.16
**Final analysis**	(*N* = 1207)	(*N* = 1401)	(*N* = 1509)
Median follow-up	52.0 months	48.0 months	29.0 months
Secondary endpoints (drug vs. placebo)	Median OS: 73.9 vs. 59.9 months; HR 0.78; 95% CI 0.64–0.96; *P* = 0.016	Median OS: 67.0 vs. 56.3 months; HR 0.73; 95% CI 0.61–0.89; *P* = 0.001	Median OS: NR vs. NR; HR 0.69; 95% CI 0.53–0.88; *P* = 0.003
	Median time to cytotoxic chemotherapy: NR vs. NR; HR 0.63; 95% CI 0.49–0.81; *P* = 0.0002	Median time to use of cytotoxic chemotherapy: NR vs. NR; HR 0.54; 95% CI 0.44–0.67	Median time to first cytotoxic chemotherapy: NR vs. NR; HR 0.58; 95% CI 0.44–0.76; *P* < 0.001
	Median time to symptomatic progression: NR vs. NR; HR 0.57; 95% CI 0.44–0.73; *P* < 0.0001	Median time to first use of new subsequent antineoplastic therapy: 66.7 vs. 19.1 months; HR 0.29; 95% CI 0.25–0.34	Median time to pain progression: 40.3 vs. 25.4 months; HR 0.65; 95% CI 0.53–0.79; *P* < 0.001
	Median time to PSA progression: 40.5 vs. 3.7 months; HR 0.07; 95% CI 0.06–0.09; *P* < 0.0001	Chemotherapy-free survival: 58.3 vs. 41.6 months; HR 0.62; 95% CI 0.52–0.72	Median time to first SSE: NR vs. NR; HR 0.48; 95% CI 0.29–0.82; *P* = 0.005
	Median time to second progression: 55.6 vs. 41.2 months; HR 0.55; 95% CI 0.46–0.66; *P* < 0.0001		
HRQoL outcomes at primary analysis (drug vs. placebo)^a^	Change from baseline in mean ± SE FACT-P total score: −0.99 ± 0.98 vs. −3.29 ± 1.97	BPI-SF (pain severity): time to progression 36.8 months vs. NR; HR 0.75; 95% CI 0.57–0.97; *P* = 0.028	BPI-SF (LSM time-adjusted AUC mean changes from baseline): pain interference: 1.1 vs. 1.3 points; difference −0.2; 95% CI −0.3 to −0.1
	Change from baseline in mean ± SE EQ VAS score: 1.44 ± 0.87 vs. 0.26 ± 1.75	FACT-P total score: time to deterioration 22.1 vs. 18.4 months; HR 0.83; 95% CI 0.69–0.99; *P* = 0.037	Pain severity: 1.3 vs. 1.4 points; difference −0.2; 95% CI −0.3 to −0.1
		EORTC QLQ-PR25: time to deterioration	FACT-P total score (LSM time-adjusted AUC mean changes from baseline): 112.9 vs. 111.6 points; difference 1.3; 95% CI 0.4–2.1
		Bowel symptoms and function: 33.2 vs. 25.9 months; HR 0.72; 95% CI 0.59–0.89; *P* = 0.0018	FACT-P PCS total score: time to deterioration 11.1 vs. 7.9 months; HR 0.80; 95% CI 0.70–0.91; *P* = 0.0005
		Hormonal treatment-related symptoms: 33.2 vs. 36.8 months; HR 1.29; 95% CI 1.02–1.63; *P* = 0.035	EORTC QLQ-PR25: time to deterioration
		Urinary symptoms: 36.9 vs. 25.9 months; HR 0.58; 95% CI 0.46–0.72; *P* < 0.0001	Bowel symptoms: 18.4 vs. 11.5 months; HR 0.78; 95% CI 0.66–0.92; *P* < 0.01
			Urinary symptoms: 25.8 vs. 14.8 months; HR 0.64; 95% CI 0.54–0.76; *P* < 0.01

*ADT* androgen-deprivation therapy, *AUC* area under the curve, *BL* baseline, *BPI-SF* Brief Pain Inventory Short Form, *C30* 30-item core questionnaire, *CI* confidence interval, *CRPC* castration-resistant prostate cancer, *CT* computerized tomography, *EORTC QLQ* European Organisation for the Research and Treatment of Cancer Quality of Life Questionnaire, *EQ VAS* European Quality of Life Visual Analogue Scale, *FACT-P* Functional Assessment of Cancer Therapy-Prostate, *FACT-P PCS* Functional Assessment of Cancer Therapy-Prostate, prostate cancer subscale, *HR* hazard ratio, *HRQoL* health-related quality of life, *LSM* least-squares mean, *mCRPC* metastatic castration-resistant prostate cancer, *MFS* metastasis-free survival, *mHSPC* metastatic hormone-sensitive prostate cancer, *mPC* metastatic prostate cancer, *MRI* magnetic resonance imaging, *nmCRPC* nonmetastatic castration-resistant prostate cancer, *nmPC* nonmetastatic prostate cancer, *NE* not estimable, *NR* not reached, *OS* overall survival, *PBO* placebo, *PC* prostate cancer, *PFS* progression-free survival, *PR25* prostate cancer-specific 25-item questionnaire, *QoL* quality of life, *rPFS* radiographic progression-free survival, *SSE* symptomatic skeletal event.

^a^Only significant results are reported in this table.

**Fig. 2:**
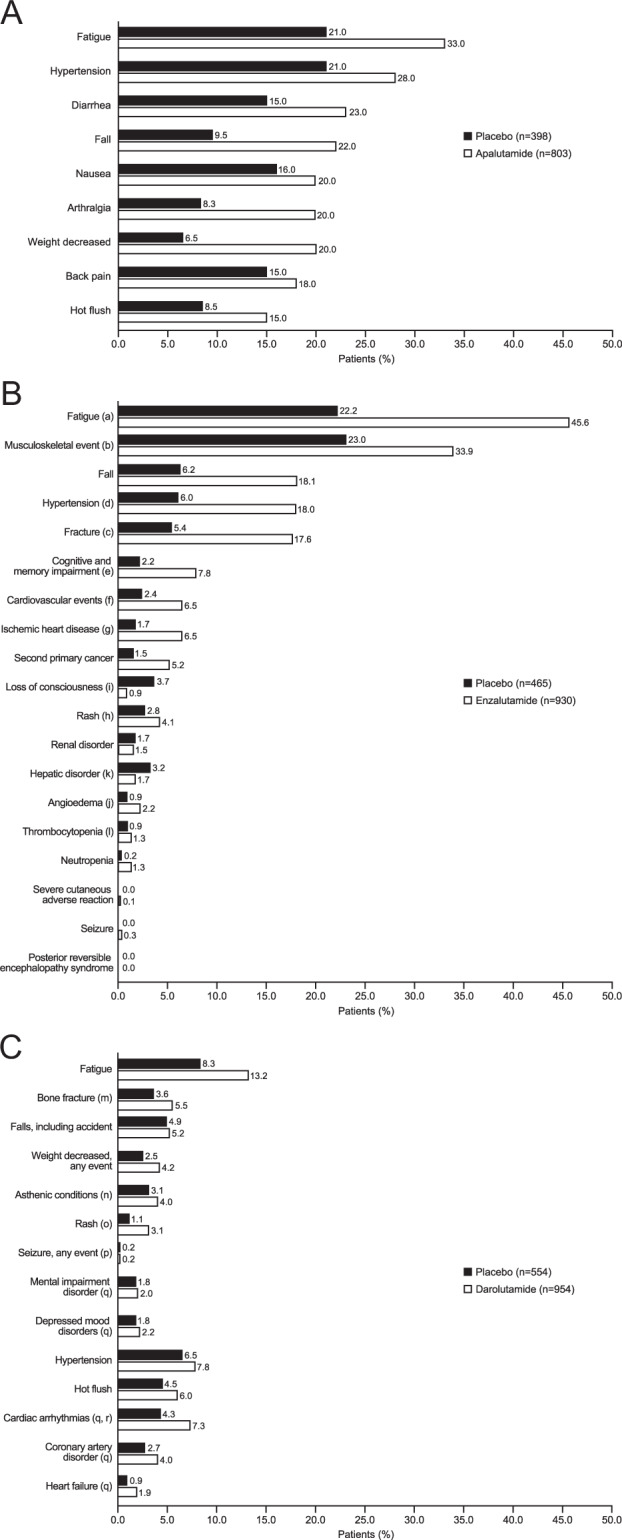
Incidence of adverse events associated with ARIs reported in the final analyses of the A SPARTAN [42], B PROSPER [45], and C ARAMIS [48] clinical trials. **A SPARTAN**: at final analysis, median follow-up was 52.0 months; median treatment duration in apalutamide arm was 32.9 months and in the placebo arm was 11.5 months. **B** **PROSPER**: at final analysis, median follow-up was 48.0 months; median treatment duration in enzalutamide arm was 33.9 months (95% CI 0.2–68.8) and in the placebo arm was 14.2 months (95% CI 0.1–51.3). ^a^Fatigue events included asthenia. ^b^Musculoskeletal events included back pain, arthralgia, myalgia, musculoskeletal pain, pain in extremity, musculoskeletal stiffness, muscular weakness, and muscle spasms. ^c^Fracture events included bone and joint injuries. ^d^Hypertension events included hypertensive retinopathy, increased blood pressure, systolic hypertension, and hypertensive crisis. ^e^Events of cognitive and memory impairment included disturbance in attention, cognitive disorders, amnesia, Alzheimer’s disease, dementia, senile dementia, mental impairment, and vascular dementia. ^f^Cardiovascular events included hemorrhagic central nervous system vascular conditions, ischemic central nervous system vascular conditions, and cardiac failure. ^g^Events of ischemic heart disease included myocardial infarction and other ischemic heart disease. ^h^Rash events included maculopapular rash, generalized rash, macular rash, papular rash, and pruritic rash. ^i^Loss-of-consciousness events included syncope and presyncope. ^j^Angioedema events included urticaria, eyelid edema, periorbital edema, swollen tongue, swollen lip, face edema, laryngeal edema, and pharyngeal edema. ^k^Hepatic disorders included hepatic failure, fibrosis, cirrhosis, and other liver damage-related conditions, and hepatitis and liver-related investigations, signs, and symptoms. ^l^Thrombocytopenia events included decreases in platelet count. **C ARAMIS**: at final analysis, median follow-up was 29.0 months; median exposure in darolutamide arm was 18.5 months and in the placebo arm was 11.6 months. ^m^Combined term comprising MedDRA terms of any fractures and dislocations, limb fractures and dislocations, skull fractures, facial bone fractures and dislocations, spinal fractures and dislocations, and thoracic cage fractures and dislocations. ^n^Combined term comprising MedDRA terms of asthenic conditions, disturbances in consciousness, decreased strength and energy, malaise, lethargy, and asthenia. ^o^Combined term comprising MedDRA terms of rash, macular rash, maculopapular rash, papular rash, and pustular rash. ^p^One additional incidence of seizure occurred in the darolutamide group during the open-label period, in a patient with a history of epilepsy. ^q^MedDRA High Level Group term. ^r^Although the incidence of cardiac arrhythmia was higher with darolutamide than with placebo, both a history of cardiac arrhythmia and electrocardiogram abnormalities were present to a greater extent in the darolutamide group at baseline, as observed at primary analysis. **ARI** androgen receptor inhibitor, **CI** confidence interval, **MedDRA** Medical Dictionary for Regulatory Activities.

The benefit of delaying disease progression or death in patients with nmCRPC should be balanced against the risk of treatment-emergent AEs. In the primary analysis of the SPARTAN trial, 96.5% and 93.2% of patients experienced an AE of any grade in the apalutamide and placebo arms, respectively [15]. The incidence of Grade 3–4 AEs was 45.1% in the apalutamide arm and 34.2% in the placebo arm. For apalutamide, fatigue, rash, falls, fractures, mental impairment, and hypothyroidism occurred more frequently than with placebo; however, when adjusted for exposure, rash was the only AE that was significantly more frequent with apalutamide compared with placebo [15]. In the final analysis of SPARTAN, the safety profile reported with apalutamide was similar to that in the primary analysis (Fig. 2A) [42]. The following Grade 3–4 AEs were higher in the apalutamide arm than in the placebo arm: hypertension and falls [42].

In terms of discontinuation of study treatment due to an AE at primary analysis, 10.6% of patients in the apalutamide arm discontinued compared with 7.0% in the placebo arm; the most common AEs leading to treatment discontinuation were rash, fatigue, sepsis, and dizziness [15]. At the time of final analysis, the discontinuation rate increased with extended treatment exposure (15.0% vs. 7.3%, apalutamide and placebo, respectively) [42].

In the primary analysis, AEs associated with death occurred in ten patients treated with apalutamide; the causes of death occurring in one patient each were acute myocardial infarction, cardiorespiratory arrest, cerebral hemorrhage, myocardial infarction, multiple organ dysfunction, and pneumonia, and the causes of death occurring in two patients each were prostate cancer and sepsis. One patient in the placebo arm died as a result of cardiorespiratory arrest [15].

While health authorities considered the safety profile of apalutamide acceptable, it bears mentioning that concomitant use of sensitive substrates of cytochrome P450 (CYP) 3A4, CYP2C19, CYP2C9, uridine 5’-diphospho-glucuronosyltransferase, P-glycoprotein, breast cancer-resistance protein (BCRP), or organic anion-transporting polypeptide 1B1 may result in loss of activity of medications such as midazolam, omeprazole, S-warfarin, fexofenadine, and rosuvastatin [40, 43].

In 2012, enzalutamide, also a second-generation oral ARI, was approved for metastatic CRPC. As of 2018, its labeled indication has been expanded to include nmCRPC based on efficacy reported in the primary analysis of the phase 3 PROSPER trial of enzalutamide versus placebo both co-administered with ADT [14, 44]. At the time of the primary analysis, median MFS was 36.6 months with enzalutamide and 14.7 months with placebo in men with high-risk nmCRPC defined by a PSADT ≤ 10 months, with a median follow-up of 18.5 and 15.1 months, in enzalutamide and placebo arms, respectively. Enzalutamide reduced the risk of radiographic progression or death by 71% compared with placebo (HR 0.29; 95% CI 0.24–0.35; *P* < 0.001) [14]. In the final analysis of the PROSPER trial (median follow-up: 48.0 months), enzalutamide was associated with significantly prolonged median OS compared with placebo (67.0 months vs. 56.3 months; HR 0.73; 95% CI 0.61–0.89; *P* = 0.001), and improvements in time to first use of new subsequent antineoplastic therapy, time to use of cytotoxic chemotherapy, and chemotherapy-free survival [45]. The PROSPER trial and its treatment outcomes are summarized in Table 1.

In PROSPER, treatment-related AEs were generally consistent with the established safety profile of enzalutamide, in that hypertension, mental impairment disorders, and major cardiovascular AEs occurred with a higher incidence than placebo. In the primary analysis, the incidence of any-grade AEs was 87 and 77% in the enzalutamide and placebo arms, and Grade 3–4 AEs occurred in 31 and 23% of patients, respectively. Compared with placebo, more patients randomized to enzalutamide reported a combined incidence of falls and nonpathologic fractures [14]. In the final analysis of PROSPER, the safety profile of enzalutamide was comparable to that at the time of primary analysis (Fig. 2B). AEs of Grade 3 or higher were reported by 48% of patients receiving enzalutamide compared with 27% receiving placebo. When adjusted for treatment exposure, falls, fatigue, and hypertension had an event rate per 100 patient-years at least 2 points higher with enzalutamide than placebo [45].

In PROSPER, 9% of patients in the enzalutamide arm discontinued treatment due to an AE compared with 6% in the placebo arm in the primary analysis, increasing to 17% and 9%, respectively, in the final analysis [14, 45]. A total of 32 patients who received enzalutamide died without evidence of radiographic progression, compared with four patients in the placebo arm. Two deaths were adjudicated to be related to enzalutamide treatment. The most common reason for death was a cardiovascular event, which was considered unrelated to the trial regimen as these deaths occurred in elderly patients with a high burden of co-existing conditions [14]. Similar to the primary analysis, the most frequent AE leading to death in the final analysis was cardiovascular events, with most patients having a previous history of cardiovascular disease [45].

Patients prescribed enzalutamide should avoid concomitant use of strong CYP2C8 inhibitors, such as gemfibrozil, and the CYP3A4 inducers, carbamazepine, phenytoin, and rifampin. In addition, enzalutamide should not be co-administered with CYP3A4, CYP2C9, and CYP2C19 substrates with a narrow therapeutic index, including, but not limited to, alfentanil, cyclosporine, warfarin, and clopidogrel [44, 46].

**Fig. 3:**
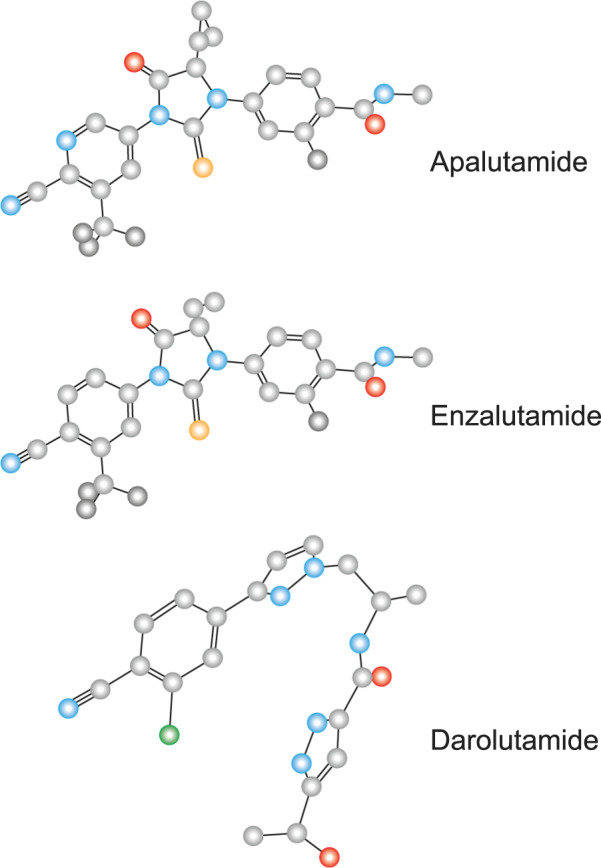
The structure of apalutamide, darolutamide, and enzalutamide. Obtained from the PubChem Open Chemistry Database.

Darolutamide is an oral, second-generation ARI, structurally distinct from enzalutamide and apalutamide (Fig. 3) [39]. It differs from apalutamide and enzalutamide in its flexible, polar structure, which is associated with low penetration of the blood–brain barrier, suggesting a low potential for incidence of central nervous system effects [39]. In 2019, the FDA approved darolutamide for the treatment of nmCRPC [47]. Approval was based on efficacy demonstrated in the primary analysis of the phase 3 ARAMIS trial of darolutamide plus ADT in patients with nmCRPC and a PSADT ≤ 10 months versus placebo plus ADT [16]. At the time of primary analysis (median follow-up: 17.9 months), darolutamide was associated with a significant increase in median MFS compared with placebo (40.4 months vs. 18.4 months, *P* < 0.001), and the risk of distant metastases or death was reduced by 59% compared with placebo (HR 0.41; 95% CI 0.34–0.50; *P* < 0.001) [16]. In the final analysis of the ARAMIS trial (median follow-up: 29.0 months), darolutamide was associated with a significant OS benefit compared with placebo, with a 31% reduction in the risk of death (HR 0.69; 95% CI 0.53–0.88; *P* = 0.003) and improvements in times to pain progression, first cytotoxic chemotherapy, and first SSE [48]. Although the number of death events required for determining OS benefit was met, the median OS was not reached in either arm due to high censoring of patients who did not experience a death event. The ARAMIS trial and its treatment outcomes are summarized in Table 1.

In the ARAMIS trial primary analysis, 83.2% and 76.9% of patients experienced an AE of any grade in the darolutamide and placebo arms, with Grade 3–4 AEs reported in 24.7% and 19.5%, respectively [16]. Darolutamide was well tolerated and no clinically relevant difference between active drug and placebo was observed for the incidence of AEs associated with ARIs, including falls, hypertension, and mental impairment [16]. The most common adverse reactions reported more frequently in the active treatment versus placebo arm of ARAMIS were fatigue, extremity pain, and rash; only fatigue had an incidence higher than 10% with darolutamide [16]. With longer follow-up time and duration of treatment in the final analysis of ARAMIS, the incidence of AEs with darolutamide remained low. In particular, the incidence of most ARI-associated AEs, such as fatigue, falls, fractures, rash, mental impairment disorders, and hypertension, continued to show minimal or no difference for darolutamide compared with placebo (Fig. 2C), which persisted when adjusted for treatment exposure [48].

Discontinuation rates due to AEs in the final analysis of the double-blind period of ARAMIS were similar in the darolutamide and placebo arms (8.9% vs. 8.7%) and remained unchanged from those at the primary analysis [16, 48]. Also, the incidence of Grade 5 AEs was similar in both treatment arms (4.0% vs. 3.4% in the darolutamide and placebo arms, respectively). Of the 37 deaths that occurred in the darolutamide arm, one was considered related to treatment (perforation of the small intestine), and of the 18 deaths in the placebo arm, two were considered related to treatment (myocardial infarction and intracranial hemorrhage) [16].

As darolutamide inhibits the BCRP transporter, its concomitant use with BCRP substrates, such as rosuvastatin, should be avoided where possible. In the event of co-administration, the treating clinician may wish to consider reducing the dose of the BCRP substrate and monitoring the patient more frequently for adverse reactions [47]. However, based on in vitro and clinical trial data, clinically relevant drug–drug interactions between darolutamide and CYP or P-glycoprotein substrates are not expected to occur [49, 50].

While second-generation ARIs have demonstrated similar efficacy for delaying metastasis and prolonging survival in pivotal clinical studies [14–16, 42, 45, 48], assessment of the benefit–risk profiles of such ARIs is vital when selecting early treatment for patients with nmCRPC in clinical practice. As this patient population is typically older and requires concomitant medications for chronic, age-related comorbidities [34, 35], appraisal of potential drug–drug interactions becomes a crucial component of individualized treatment decision-making, in addition to tolerability and safety profiles.

## Current treatment guidelines

In the pharmacologic management of nmCRPC, patient–provider-shared decision-making should be based on the likelihood of disease progression, the presence of chronic comorbidities, the risk–benefit profile of available ARIs, and drug safety. Current options for additional hormonal therapy in nmCRPC focus on the blockade of androgen synthesis or activity [51].

Following recent updates, guidelines from the NCCN and EAU now recommend that patients with nmCRPC and a PSADT ≤ 10 months who are at increased risk of disease progression be treated with enzalutamide, apalutamide, or darolutamide, in addition to continuing ADT, to delay metastasis [4, 8]. It is anticipated that the American Urological Association, the Canadian Urological Association, and Japanese Urological Association also will include darolutamide in their updated guidelines for the treatment of high-risk patients with nmCRPC [52–54]. Guideline recommendations are based on high-level evidence of efficacy, which all three ARIs demonstrated in their respective phase 3 clinical trials with MFS as the primary efficacy endpoint. While OS is the conclusive endpoint for treatment efficacy [55], MFS was considered a sufficiently strong surrogate of OS for the regulatory approval of apalutamide, enzalutamide, and darolutamide [56–58]. The recently reported OS data for these three second-generation ARIs have now confirmed that MFS is a reliable and robust surrogate endpoint and have further strengthened the recommendation to prescribe these agents to patients with nmCRPC.

While first-generation ARIs, such as flutamide, bicalutamide, and nilutamide are included in the US, European, and Canadian guidelines, no high-level evidence for a survival benefit has been presented in patients with nmCRPC [4, 8, 52, 53]. According to the 2018 American Urological Association guideline amendment for CRPC, in selected patients with nmCRPC at high risk of developing metastases and who refuse or cannot tolerate one of the standard therapies and are unwilling to accept observation on continuous ADT, the androgen synthesis inhibitor abiraterone acetate, in combination with prednisone, also may be offered [52, 59, 60].

## Maintaining quality of life in patients with nmCRPC

The patient with a rising PSA level and no radiologic evidence of metastases is usually asymptomatic from his disease but may exhibit symptoms related to age, chronic, nonmalignant comorbidities, long-term concomitant medications, adverse effects associated with ADT, and drug–drug interactions. Consequently, a consistent goal of initiating pharmacotherapy in nmCRPC should be to delay time to metastases while preserving quality of life as close to pretreatment status as possible [29].

Second-generation ARIs prolong survival while maintaining HRQoL in patients with nmCRPC. In clinical trials of the three approved ARIs, patient-reported outcomes captured by validated, self-administered questionnaires indicated that no treatment-induced deterioration in quality of life occurred, and that in some instances, improvement was observed in prostate cancer-specific domains [14–16].

In the SPARTAN trial, the least-squares mean change from baseline showed that HRQoL deterioration was more apparent with placebo compared with apalutamide [61]. In PROSPER, a trend favoring enzalutamide was observed for all domains of the Functional Assessment of Cancer Therapy-Prostate, with the exception of physical well-being [62]. In ARAMIS, time to deterioration of European Organisation for Research and Treatment of Cancer Quality of Life Questionnaire-Prostate Cancer Module (EORTC QLQ-PR25) outcomes demonstrated statistically and clinically significant delays with darolutamide versus placebo for urinary symptoms (25.8 vs. 14.8 months; HR 0.64; 95% CI 0.54–0.76; *P* < 0.01) [63].

Thus, these three second-generation ARIs may offer patients with nmCRPC a therapeutic option that addresses the need to prevent disease progression while maintaining quality of life.

## Conclusions

Clinical development in the field of nmCRPC is evolving rapidly. The second-generation ARIs, apalutamide, enzalutamide, and darolutamide, have revolutionized the treatment landscape for nmCRPC. All three ARIs have demonstrated significant prolongation of MFS, and the recently reported final analyses of the SPARTAN, PROSPER, and ARAMIS trials indicate a significant OS benefit in patients with nmCRPC [14–16, 42, 45, 48].

Although second-generation ARIs have acceptable tolerability and maintain quality of life in patients with nonmetastatic disease, their individual safety profiles and potential for drug–drug interactions with concomitant medications should be considered. The benefit–risk profile of ARIs in patients with nmCRPC is an important clinical consideration and therapies that do not compound ADT-related AEs or contribute to additional therapeutic burden due to drug–drug interactions may be preferred [37].

In summary, while delaying the onset of metastasis and ultimately prolonging survival represents the central objective of pharmacotherapy, appropriate treatment of patients with nmCRPC must strike an individualized balance between clinical benefit and potential risk [37].
